# Matrix Metalloproteinases in Human Decidualized Endometrial Stromal Cells

**DOI:** 10.3390/cimb43030146

**Published:** 2021-11-26

**Authors:** Yoji Hisamatsu, Hiromi Murata, Hiroaki Tsubokura, Yoshiko Hashimoto, Masaaki Kitada, Susumu Tanaka, Hidetaka Okada

**Affiliations:** 1 Department of Obstetrics and Gynecology, Kansai Medical University, Osaka 573-1010, Japan; hisamayj@kouri.kmu.ac.jp (Y.H.); murathir@hirakata.kmu.ac.jp (H.M.); tsubokuh@hirakata.kmu.ac.jp (H.T.); hashiyos@hirakata.kmu.ac.jp (Y.H.); 2 Department of Anatomy, Kansai Medical University, Osaka 573-1010, Japan; masaaki.kitada@gmail.com

**Keywords:** MMP: matrix metalloprotease, TIMP: tissue inhibitor of metalloproteinase, HAND2: heart and neural crest derivatives expressed 2, SNAI1: snail family transcriptional repressor 1

## Abstract

Cyclic changes, such as growth, decidualization, shedding, and regeneration, in the human endometrium are regulated by the reciprocal action of female hormones, such as estradiol (E_2_), and progesterone (P_4_). Matrix metalloproteases (MMPs) and tissue inhibitors of MMPs (TIMPs) control the invasion of extravillous trophoblast cells after implantation. Several MMPs and TIMPs function in the decidua and endometrial stromal cells (ESCs). Here, we aimed to systematically investigate the changes in MMPs and TIMPs associated with ESC decidualization. We evaluated the expression of 23 MMPs, four TIMPs, and four anti-sense non-coding RNAs from MMP loci. Primary ESC cultures treated with E_2_ + medroxyprogesterone acetate (MPA), a potent P_4_ receptor agonist, showed significant down-regulation of *MMP3*, *MMP10*, *MMP11*, *MMP12*, *MMP20*, and *MMP27* in decidualized ESCs, as assessed by quantitative reverse transcription PCR. Further, *MMP15* and *MMP19* were significantly upregulated in decidualized ESCs. siRNA-mediated silencing of Heart and Neural Crest Derivatives Expressed 2 (HAND2), a master transcriptional regulator in ESC decidualization, significantly increased *MMP15* expression in untreated human ESCs. These results collectively indicate the importance of *MMP15* and *MMP19* in ESC decidualization and highlight the role of HAND2 in repressing *MMP15* transcription, thereby regulating decidualization.

## 1. Introduction

In the human endometrium, growth, decidualization, shedding, and regeneration occur repeatedly with each menstrual cycle, even without embryo implantation [1,2]. These events are controlled by the reciprocal action of estradiol (E_2_) and progesterone (P_4_), the major ovarian steroid hormones [3]. Human endometrial stromal cells (ESCs) are decidualized spontaneously by endometrial changes during the secretory phase [4]. ESC decidualization is essential to provide a healthy environment for the embryo before placentation, protecting the embryo from maternal immune rejection [5,6].

Upon implantation, placental extravillous trophoblast (EVT) cells have been shown to invade through the decidua and migrate towards the spiral arteries of the placenta [7]. EVT cells first invade the uterine epithelium, then the basement membrane, followed by the extracellular matrix (ECM), and finally the decidual stroma [8]. Matrix metalloproteases (MMPs), controlled by tissue inhibitors of MMPs (TIMPs), play a notable role in regulating the invasion potential of EVTs by degrading ECM components. Several MMPs and TIMPs are known to be expressed in the decidua and ESCs [9]. ESCs and decidualized ESCs undergo a cycle of endometrial breakdown and regeneration and decidua remodeling by secreting various MMPs that degrade ECM components and activate growth factors [10,11]. MMPs belong to a group of structurally related zinc-containing endopeptidases. MMPs degrade and remodel specific components of the ECM. In total, 23 types of human MMP gene families with various vital functions have been identified to date [12]. Further, TIMPS regulate MMP activity by processing pro-zymogens and inhibiting active enzymes [13].

Increasing studies have highlighted the role of MMPs in the endometrium; however, a systematic investigation of the changes in MMPs and TIMPs associated with ESC decidualization is lacking. In this study, we examined the expression of 23 MMPs, four non-coding RNAs from MMP loci (*MMP2-AS1*, *MMP23A*, *MMP24OS*, and *MMP25-AS1*), and four TIMPs in control and decidualized human ESCs induced by E_2_ + medroxyprogesterone acetate (MPA), a synthetic progestin that acts as a potent P_4_ receptor agonist [14]. Our findings indicate that many MMPs are significantly downregulated, whereas *MMP15* and *MMP19* are significantly upregulated during the decidualization of ESCs. 

## 2. Materials and Methods

### 2.1. Ethics Statement

Explanations were given orally and in written form, and informed consent was obtained in written form from each patient. This study was performed with approval from the review board of Kansai Medical University (protocol number 2006101). This study also adheres to the principles of the Declaration of Helsinki.

### 2.2. ESCs Collection

Human endometrial tissues from 11 patients aged 42–50 years with regular menstrual cycles were obtained (Table 1). These patients underwent hysterectomies without preoperative hormonal therapy. Uterine histological examinations revealed myomas, which are benign tumors. All endometrial specimens were confirmed to be histologically normal. ESCs were then immediately purified from endometrial tissues as described below.

### 2.3. Human ESC Culture and Treatment with Steroid Hormones

To purify human ESCs from endometrial tissues, a standard enzyme digestion method was used as described previously [15]. Purified ESCs were cultured in phenol red-free DMEM/F12 medium (Thermo Fisher Scientific, Waltham, MA, USA) with 2 mmol/L Glutamax (Thermo Fisher Scientific), 10% dextran-coated charcoal-stripped fetal calf serum (Thermo Fisher Scientific), 100 μg/mL streptomycin, and 100 IU/mL penicillin at 37 °C under a humidified atmosphere of 5% CO_2_ in air. Phenol red-free DMEM/F12 medium and dextran-coated charcoal-stripped fetal calf serum were used to remove the effects of endogenous steroid hormones. The culture media were exchanged every three days. ESCs were used for the experiments when they were nearly confluent. Immunohistochemical analysis for vimentin was performed to confirm ESC purity, and >99% vimentin-positive cells were detected in confluent ESCs as described previously [15]. For experiments, ESCs were seeded in a 6-well plate and allowed to reach confluence before treatment. Confluent cultures of ESCs were treated with E_2_ (10^−8^ mol/L) and medroxyprogesterone acetate (MPA) (10^−7^ mol/L) (E_2_ + MPA treatment) for up to 12 days to induce decidualization, as per a previously established method [14,16]. Untreated cells were used as controls.

### 2.4. siRNA-Meditated HAND2 Silencing

HAND2 silencing was performed by reusing the samples prepared in a previous study [14,17]. Briefly, ESCs were transfected with 10 nmol/L of two different siRNAs targeting the human HAND2 gene (HAND2-1; catalog no. HSS145155 and HAND2-2; HSS190355) or a non-silencing RNA (catalog no. HSS12935–112) using Lipofectamine RNAiMAX reagent (Thermo Fisher Scientific) on day 0 and were then cultured for 5 days. Silencing of *HAND2* was verified by RT-qPCR and Western blotting in a previous study [17]. Experiments were repeated at least four times using cell preparations drove from different patients.

### 2.5. Quantitative Reverse Transcription PCR

Total RNA was extracted from cultured ESCs with/without E_2_+MPA treatment for twelve days using the RNeasy Mini kit (Qiagen, Venlo, The Netherlands). cDNA was reverse-transcribed with a ReverTra Ace™ qPCR RT master mix with gDNA remover (TOYOBO, Osaka, Japan). Quantitative PCR was then performed with a Rotor-Gene platform (Qiagen) and the Thunderbird qPCR Mix (Toyobo). Primer sequences corresponding to each gene used for PCR are shown in Table 2. The 2-ΔΔCt method was used to calculate relative target gene expression levels [18] using Elongation factor-1α (*EF1A)* as a housekeeping gene [19]. PCR with all cDNA samples was performed in duplicate.

### 2.6. Immunoblot Analysis

Total soluble protein was purified from human ESCs that were cultured with/without E_2_ + MPA treatment for twelve days using Mammalian Protein Extraction Reagent (Thermo Fisher Scientific) and a protease inhibitor cocktail (Nacalai Tesque, Osaka, Japan). Immunoblotting was performed to analyze MMP15 protein levels. Soluble proteins were loaded onto 10% sodium dodecyl sulfate-polyacrylamide gel electrophoresis (SDS-PAGE gel), electrotransferred to a polyvinylidene diflouride membrane, and blocked with Blocking One (Nacalai Tesque). The membrane was incubated overnight at 4 °C with Rabbit Anti-MMP-15 antibody (1:1000; Sigma-Aldrich, Tokyo, Japan, Cat# SAB4501903, RRID: AB_10746129) or β-actin mouse antibody (1:5000; Sigma-Aldrich, Cat# A5316, RRID: AB_476743) in 5% Blocking One in TBS containing 0.1% Tween-20. For the secondary antibody, goat anti-rabbit peroxidase-labeled IgG (H+L) (1:5000; VECTOR laboratories, Cat# PI-1000, RRID: AB_2336198) or sheep peroxidase-linked anti-mouse IgG antibody (1:10,000; GE Healthcare Life Science, Cat# NA931, RRID: AB_772210) was used. ECL Prime Western Blotting Detection Reagent (GE Healthcare Life Science) was used to visualize the resulting complexes. LAS 4000 (GE Healthcare Life Science) was used to detect the bands, protein band intensities were analyzed using ImageJ [20], and MMP15 mounts were normalized to β-actin levels.

### 2.7. Statistical Analyses

The Shapiro–Wilk normality test showed normal distribution in each group. Comparisons of MMP and TIMP levels between the control and E_2_ + MPA groups were performed using Welch’s *t*-test. siRNA effects were compared using Welch’s *t*-test with/without Bonferroni correction. All values were two-sided with statistical significance set at a *p* value < 0.05. Statistical analyses were performed using IBM SPSS Statistics version 21.0 (IBM Corp., Armonk, NY, USA).

## 3. Results

### 3.1. Expression Changes in MMPs

**Figure 1:**
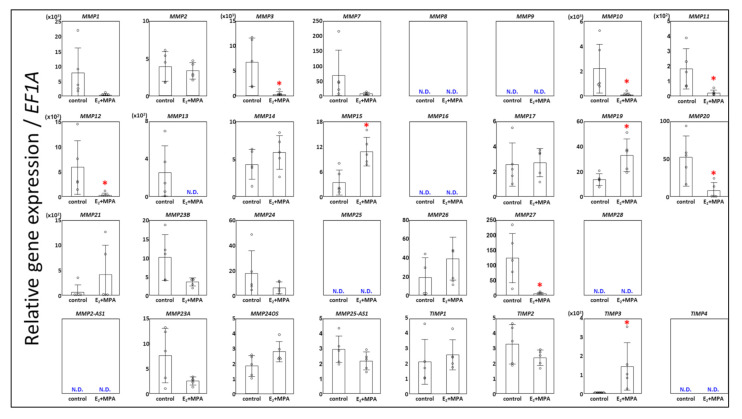
Effect of E_2_ + MPA treatment on MMPs or TIMPs in human endometrial stromal cells. Human endometrial stromal cells were treated with E_2_ (10^−8^ mol/L) + MPA (10^−7^ mol/L) (E_2_ + MPA) or without hormonal treatment (control) for 12 days. The expression levels of all protein-coding MMPs, TIMPs, and long non-coding antisense RNAs from MMP loci were analyzed by quantitative RT-PCR. MMP and TIMP RNA levels were normalized to those of EF1A. Values are shown as mean fold change ± SD of independent experiments using cells derived from different patients (*n* = 5). Each dot indicates the value for different cell preparations. An asterisk indicates statistically significant differences from the control group, * *p* < 0.05.

**Figure 2:**
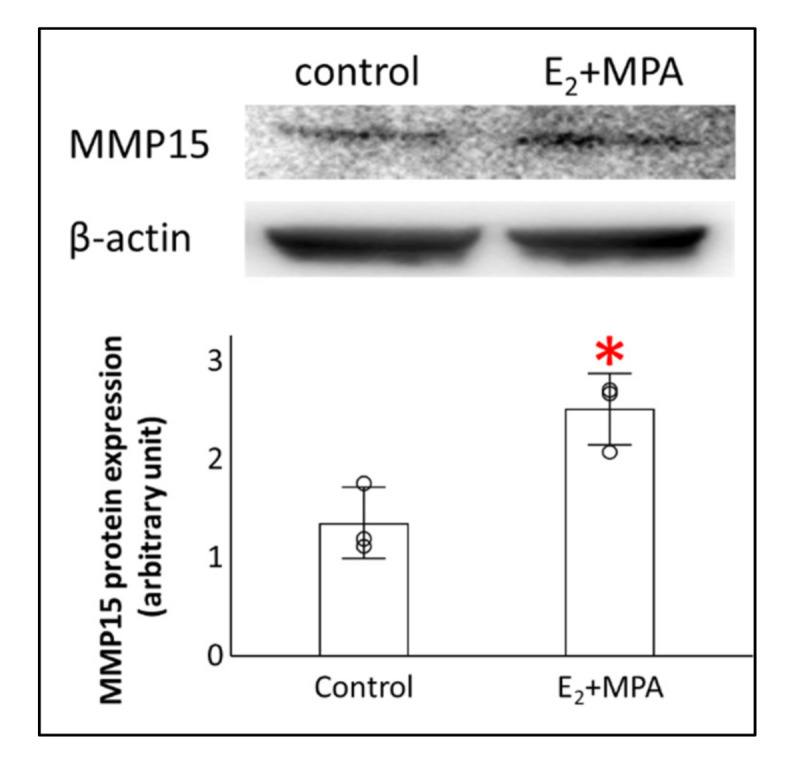
Effect of E_2_ + MPA treatment on MMP15 proteins in human endometrial stromal cells. Human endometrial stromal cells were treated with E_2_ (10^−8^ mol/L) + MPA (10^−7^ mol/L) (E_2_ + MPA) or without hormonal treatment (control) for 12 days. The protein expression levels of MMP15 and β-actin were analyzed by immunoblot analysis. MMP15 were normalized to those of β-actin. Values are shown as mean fold change ± SD of independent experiments using cells derived from different patients (*n* = 3). Each dot indicates the value for different cell preparations. An asterisk indicates statistically significant differences from the control group, * *p* < 0.05.

First, we investigated the gene expression profile of control ESCs or ESCs treated with E_2_ + MPA for 12 days to investigate the involvement of MMPs in the decidualization process. Our analysis revealed that *MMP3*, *MMP10*, *MMP11*, *MMP12*, *MMP20,* and *MMP27* were significantly downregulated in decidualized ESCs compared with those in the control (Figure 1). *MMP1*, *MMP7*, *MMP13*, and *MMP23B* tend to have lower expression in decidualized ESCs than in the control but did not show significant down-regulation due to variability among samples. Moreover, *MMP15* and *MMP19* were significantly upregulated in decidualized ESCs compared to those in the control (Figure 1), with no significant differences in *MMP2*, *MMP7*, *MMP14*, *MMP17*, *MMP21*, *MMP24*, and *MMP26* between the two groups. MMP15 protein expression in ESCs was also confirmed (Figure 2). Furthermore, as with gene expression, E_2_ + MPA treatment significantly increased the amount of MMP15 protein (Figure 2).

Although all genes were amplified from the endometrial cDNA pool from cells in the proliferative to secretory phase, the CT values in the RT-qPCR analysis of *MMP8*, *MMP9*, *MMP16*, *MMP25*, and *MMP28* in both decidualized and control ESCs were about the same levels as the CT value of the no-template control (with only distilled water, negative control). Therefore, we hypothesized that *MMP8*, *MMP9*, *MMP16*, *MMP25*, and *MMP28* are not expressed in ESCs (Figure 1). 

It is well known that antisense long non-coding RNAs are involved in the expression of protein-coding genes at their loci [21]. Thus, we examined the expression of *MMP2-AS1*, *MMP23A*, *MMP24OS*, and *MMP25AS-1* to find a novel mechanism regulating MMP expression in ESCs by antisense long non-coding RNAs. However, we did not detect *MMP2-AS1* expression in ESCs or find any involvement of *MMP23A*, *MMP24OS*, and *MMP25AS-1* in decidualization (Figure 1).

### 3.2. Expression Chages in TIMPs

TIMP3 was significantly increased in decidualized ESCs (Figure 1) as in our previous studies [19]. However, no significant differences in TIMP1, TIMP2, and TIMP4 expression were detected between E_2_ + MPA and the control group.

### 3.3. Potential Transcription Factors for MMP15

**Figure 3:**
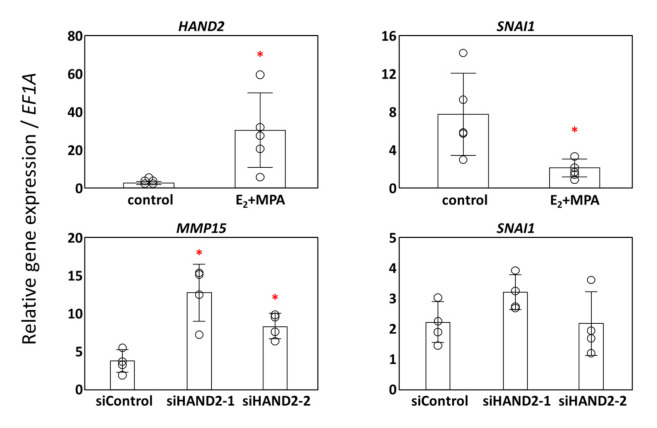
Effect of siRNA-mediated HAND2 silencing on the expression of *MMP15* or *SNAI1* in human endometrial stromal cells. Human endometrial stromal cells (ESCs) were treated with E_2_ (10^−8^ mol/L) + MPA (10^−7^ mol/L) or without hormonal treatment (control) for 12 days. HAND2 and SNAI1 were analyzed by quantitative RT-PCR (*n* = 5, upper panels). ESCs were transfected with human HAND2 siRNA (HAND2-1 and HAND2-2) or a non-silencing RNA on day 0 and then cultured for 5 days without hormonal treatment (lower panels). *MMP15* and *SNAI1* were quantified using quantitative RT-PCR and corrected for *EF1A* (lower panels). The bars show the mean of relative gene expression ± SD of independent experiments using cells derived from different patients (*n* = 4). Each dot indicates the value for cells derived from different patients. An asterisk indicates statistically significant differences from the control group, * *p* < 0.05.

Progesterone receptor (PGR) is known to bind to the *MMP19* genomic locus and to regulate *MMP19* expression during decidualization [22], whereas the transcriptional regulation of *MMP15* during decidualization is not well understood. Therefore, we searched the ChIP-Atlas database (https://chip-atlas.org/, accessed on 23 April 2021) for transcription factors that may regulate the transcription of *MMPs* [23]. We found that HAND2, a master transcriptional regulator [24,25], which is upregulated in decidualization of ESCs, may bind to the upstream region of *MMP2* and *MMP15* genes. There are no ChIP-based studies that report the binding of HAND2 to the upstream region of other *MMPs* or to that of any of the *TIMPs* in the ChIP-Atlas database. Moreover, *MMP15* is regulated by SNAI1, a transcriptional repressor, and *SNAI1* is regulated by HAND2 in the heart [26,27]. Therefore, we examined the gene expression changes of *HAND2* and *SNAI1* during the decidualization of ESCs to understand their involvement in the regulation of *MMP15* expression. As reported in our previous study [25], *HAND2* expression was significantly upregulated in ESCs with E_2_ + MPA treatment compared with that in the control group (Figure 3). In contrast, *SNAI1* expression was significantly suppressed in ESCs with E_2_ + MPA treatment (Figure 3). Therefore, we examined the expression of *SNAI1* and *MMP1*5 in siRNA-mediated *HAND2-*silenced samples from our previous study [14]. We found upregulation of MMP15 expression upon silencing of *HAND2* (Figure 3), suggesting a crucial role of HAND2 in regulating the baseline of *MMP15* expression in ESCs. However, *SNAI1* expression was not affected by the decrease in the HAND2 expression (Figure 3), thereby suggesting that *SNAI1* expression in ESCs was independent of HAND2.

## 4. Discussion

In this study, we surprisingly found significant downregulation of many MMPs and significant upregulation of *MMP15* and *MMP19* during ESC decidualization. Moreover, consistent with findings from our previous study [19], only *TIMP3* expression was significantly increased among TIMP family members. Such observations collectively led us to conclude that the downregulation of many *MMPs* and up-regulation of *MMP15*, *MMP19*, and *TIMP3* must be necessary for ESC decidualization.

To check the purity of the patient-derived ESCs after isolation, we confirmed vimentin expression on IHC staining and elevation of *IGFBP1* and *PRL*, which are known as decidualized-ESCs markers. Furthermore, proof that other endometrial mesenchymal cells were eliminated in this study derives from the following: (1) *MMP9* has been reported to be upregulated in umbilical cord-derived mesenchymal stem cells in uterine scars [28], whereas *MMP9* was not expressed in our study; (2) *MMP16* is known to be expressed in endometrial mesenchymal stem cells [29] but was not found in our study; and (3) *MMP19* is known to show lower expression in endometrial mesenchymal stem cells than in glandular cells [29], but *MMP19* expression was found to be elevated in our study. Therefore, the influence of mesenchymal stem cells is considered negligible in this study.

MMP15 is the only membrane type (MT)-MMP expressed in the human placenta during early pregnancy [30,31,32]. MT-MMPs including MMP15, are specifically expressed in the front line of invading cells and can degrade ECM in a site-specific manner during cell invasion in cancer [33]. Moreover, cytotrophoblasts (CTB) from human decidual tissue show significant *MMP15* expression [9]. Majali-Martinez A et al. also showed that stromal CTBs in the decidua basalis of the first trimester are the primary source of MMP15 [34]. Therefore, we hypothesized that decidualized ESCs might use MMP15, together with stromal CTBs, to break MMP15-specific substrates, including several decidual ECM proteins such as collagen I and IV, laminin, and vitronectin [35,36] for CTB invasion.

We found a possibility that HAND2, a master transcriptional regulator [24,25] upregulated in ESC decidualization, may bind to the upstream regions of *MMP2* and *MMP15* genes with analysis of the ChIP-Atlas database (https://chip-atlas.org/, accessed on 23 April 2021) [23]. There are no ChIP reports on HAND2 binding to the upstream regions of *MMPs* without *MMP2* and *MMP15* and *TIMPs* in the ChIP-Atlas database. Similarly, the transcriptional regulator FOXO1, which is upregulated in decidualization [17,37,38,39], was found to not bind to the upstream region of any *MMP,* including *MMP2* and *MMP15* or *TIMPs* from the Chip-Atlas search. The consensus motif for HAND2 binding (CAGATG) [40] is found in two locations (−1253/−1248, −1523/−1518) of the 1.5-kilobase region upstream of the human *MMP15* gene. These sequences appear to be vital because they are conserved even in the proximal upstream region of the mouse *MMP15* gene (−1472/−1467, −1636/−1631). However, the HAND2 consensus motif (CAGATG) was absent even in the 5-kilobase upstream region of the human *MMP2* gene. In contrast, the results of siRNA-mediated HAND2 silencing showed HAND2 repressed *MMP15* transcription, suggesting that a group of other transcription factors increased in ESCs under the influence of P_4_ upregulate *MMP15* transcription. Subsequently, the increased amount of MMP15 protein might promote menstruation by cleaving proMMP2 to the active form, MMP2, which is compatible with many substrates [41,42]. *Mmp15* is known to be regulated by the P_4_ receptor and the CCAAT/enhancer-binding protein β (Cebpb) in medaka follicles [43]. CEBPBs are known regulators affecting the proliferation and differentiation of ESCs [44]. We applied TFBIND [45] used in a study of the medaka genome [43] to the 2 kb upstream region of the human *MMP15* gene; however, unlike in the medaka, a putative P_4_ receptor motif was not found in the human counterpart. Moreover, multiple putative CEBPBs motifs were found in the 2k upstream region of the human *MMP15* gene (−194/183, −164/−155, −151/−140), suggesting that CEBPB might be implicated in the transcriptional regulation of human *MMP15*. Interestingly, we found putative estrogen receptor 1 binding motifs in the 2k upstream region of the human *MMP15* gene (−85/−67). The involvement of these factors in regulating *MMP15* expression in ESCs needs further investigation to clearly delineate the mechanistic basis of the regulation.

MMP15 expression is also known to be driven by SNAI1, an epithelial-mesenchymal transition (EMT)-related transcription factor [46] in endocardial cushion development [27]. SNAI1 is also known to be transcriptionally regulated by HAND2 [26]. E-box sequence-mediated regulation by SNAI1 is conserved between humans and mice [27]. In contrast to endocardial development, SNAI1 might suppress the expression of *MMP15* in ESCs, according to many reports showing how the same DNA binding factors regulate the transcription of several mRNAs differentially during the developmental stages [47]. A similar phenomenon depending on cell type is supposed to occur between *MMP15* expression and binding of SNAI1 to the motif in the promoter region of *MMP15* gene. Our observations from siRNA-mediated *HAND2* silencing experiments also support that there is no direct relationship between *SNAI1* transcription and HAND2 in ESCs. Therefore, we propose SNAI1-mediated *MMP15* regulation to be independent of HAND2 in ESCs. Interestingly, in the process of decidualization, MMP15 itself is a regulator of EMT [48,49]. Furthermore, as *MMP15* is upregulated in preeclampsia [49], proper regulation of MMP15 in decidualized ESCs could be necessary for normal pregnancy. 

It is known that progesterone receptor (PGR) binds to the *MMP19* genomic locus and regulates *MMP19* expression during decidualization [22]. Angiogenesis is the process by which endothelial cells migrate and invade through the ECM by inducing angiogenesis-promoting secretory factors, and new blood vessels are formed from existing ones. MMP19 localizes in vascular endothelial and smooth muscle cells [50] and exerts anti-angiogenic effects on endothelial cells by generating angiostatin-like fragments [51]. These angiostatin-like fragments stabilize blood vessels by inhibiting the proliferation of human microvascular endothelial cells and reducing the formation of capillary-like structures [51]. Similarly, PGR-induced MMP19, which decreases in low fertility animals, is considered necessary in maintaining vascular stability during decidualization, which may be critical for pregnancy [52,53].

In this study, we found downregulated expression of multiple MMPs in association with decidualization. Interestingly, a relationship between recurrent spontaneous abortion and *MMP10* mutation is known [54]. Therefore, we hypothesized that MMP10 in ESCs might be essential for embryo receptivity.

The family of TIMPs has four non-redundant members in the human genome to physiologically inhibit MMPs. The balance between MMP and TIMP activity is vital for the normal function and stability of the ECM. TIMP3 is the only TIMP family member with a high affinity for ECM proteoglycans, and the widest range of substrates, including all MMPs, disintegrins, and metalloproteinases (ADAMs), with thrombospondin motifs [55,56,57,58,59,60,61]. Therefore, we hypothesized that TIMP3 has more diverse functions beyond MMP inhibition, such as regulating MMPs during decidualization. TIMP3 has been reported to have MMP-independent functions with a variety of interactors [62,63,64,65,66,67,68,69]. For example, TIMP3 is known to inhibit the binding of VEGF to VEGFR2 [62], thereby increasing vascular stability, as TIMP3 inhibits vascular endothelial cell proliferation and migration, and increases vascular permeability caused by VEGF–VEGFR2 interaction.

## 5. Conclusions

In conclusion, from the results of our study, we established an association between *MMP15* expression and decidualization. However, as we could not prove whether *MMP15* elevation is a result of decidualization or whether it is involved in decidualization itself, further studies of *MMP15* knockdown in ESCs are necessary to understand the mechanistic details of MMP15 function.

## Figures and Tables

**Table 1:** Sample Information.

Sample No.	Materials	Methods		Age	Menstrual Cycle Phase at the Time of Collection
1	Primary culture ESCs	Treated with E_2_ + MPA for 12 days	RT-qPCR	50	Proliferative
2	Primary culture ESCs	Treated with E_2_ + MPA for 12 days	RT-qPCR	45	Mid-secretory
3	Primary culture ESCs	Treated with E_2_ + MPA for 12 days	RT-qPCR	48	Late-secretory
4	Primary culture ESCs	Treated with E_2_ + MPA for 12 days	RT-qPCR	50	Mid-secretory
5	Primary culture ESCs	Treated with E_2_ + MPA for 12 days	RT-qPCR	44	Late-secretory
6	Primary culture ESCs	Transfection of HAND2-siRNA	RT-qPCR	49	Mid-secretory
7	Primary culture ESCs	Transfection of HAND2-siRNA	RT-qPCR	46	Late-secretory
8	Primary culture ESCs	Transfection of HAND2-siRNA	RT-qPCR	42	Proliferative
9	Primary culture ESCs	Transfection of HAND2-siRNA	RT-qPCR	47	Late-secretory
10	Primary culture ESCs	Treated with E_2_ + MPA for 12 days	Immunoblot analysis	45	Mid-Secretory
11	Primary culture ESCs	Treated with E_2_ + MPA for 12 days	Immunoblot analysis	42	Proliferative
12	Primary culture ESCs	Treated with E_2_ + MPA for 12 days	Immunoblot analysis	49	Proliferative

**Table 2:** Oligonucleotides.

MMP1	MMP1_884F	5′-ACAAACCCCAAAAGCGTGTG-3′
	MMP1_993R	5′-AGAAGGGATTTGTGCGCATG-3′
MMP2	MMP2_697F	5′-ACAGTGGATGATGCCTTTGC-3
	MMP2_808R	5′-AGCGGCCAAAGTTGATCATG-3′
MMP3	MMP3_1578F	5′-TTCGTTTTCTCCTGCCTGTG-3′
	MMP3_1693R	5′-AGCAGCAGCCCATTTGAATG-3′
MMP7	MMP7_362F	5′-TTCCAAAGTGGTCACCTACAGG-3′
	MMP7_459R	5′-TGCCCCACATGTTTAAAGCC-3′
MMP8	MMP8_355F	5′-ATGAAAAAGCCTCGCTGTGG-3′
	MMP8_436R	5′-AGTTAGTGCGTTCCCACTTG-3′
MMP9	MMP9_1560F	5′-ATGCCTGCAACGTGAACATC-3′
	MMP9_1646R	5′-AGAATCGCCAGTACTTCCCATC-3′
MMP10	MMP10_373F	5′-CCTTACATACAGGATTGTGAATTATACACC-3′
	MMP10_519R	5′-GAGATCATTATATCAGCCTCTCCTTCATAC-3′
MMP11	MMP11_558F	5′-ATGCCTTCTTCCCCAAGACTC-3′
	MMP11_684R	5′-AGCACGTGGCCAAATTCATG-3′
MMP12	MMP12_440F	5′-ACGCAATCCGGAAAGCTTTC-3′
	MMP12_527R	5′-ACCAAAATGTCAGCCATGCC-3′
MMP13	MMP13_864F	5′-AACGCCAGACAAATGTGACC-3′
	MMP13_991R	5′-AAAACAGCTCCGCATCAACC-3′
MMP14	MMP14_461F	5′-AACAGGCAAAGCTGATGCAG-3′
	MMP14_567R	5′-AGCGCTTCCTTCGAACATTG-3′
MMP15	MMP15_1187F	5′-TCATGGTACTCTTTGCCTCTGG-3′
	MMP15_1310R	5′-TCTGCGTCAAAATGGGTGTC-3′
MMP16	MMP16_654F	5′-AATGGCAGCACAAGCACATC-3′
	MMP16_748R	5′-ATCAAAGGCACGGCGAATAG-3′
MMP17	MMP17_1095F	5′-ATGCAGCACTCACTTTGACG-3′
	MMP17_1169R	5′-CGCCAGAAGTACTTGCCTTTG-3′
MMP19	MMP19_1606F	5′-AGCCACAGAAACCACGTTTG-3′
	MMP19_1698R	5′-AAATGAAAGGGTGGGTGGTG-3′
MMP20	MMP20_1053F	5′-TGGATGCAGCTTACGAAGTG-3′
	MMP20_1195R	5′-TATTTGCTGCACGTGCCTTG-3′
MMP21	MMP21_889F	5′-ACGGGATCCATAATGCAACC-3′
	MMP21_1025R	5′-TTGCGAATCCAGTCAAACGC-3′
MMP23B	MMP23B_1021F	5′-CCTCCACAAGAAAGGGAAAGTG-3′
	MMP23B_1151R	5′-TGTAGGTGCCCTCATTGACG-3′
MMP24	MMP24_847F	5′-AGGAAATGCCAACCATGACG-3′
	MMP24_990R	5′-AGCTTGAAGTTGTGCGTCTC-3′
MMP25	MMP25_3278F	5′-TGGCTGTTTCGTGGCATTTC-3′
	MMP25_3407R	5’-TGGACAGCAACTTAGGAAGTGG-3′
MMP26	MMP26_1127F	5′-AAAGCACTAGAGCAGCCTTG-3′
	MMP26_1215R	5′-AGCGTTTTGAGTGTCGGTTC-3′
MMP27	MMP27_853F	5′-ACCTGCTAAGCCAAAGGAAC-3′
	MMP27_932R	5′-TGCGGAAAGTTGTGATAGCG-3′
MMP28	MMP28_1214F	5′-AAACGCAGGGCCCTAAATAC-3′
	MMP28_1283R	5′-TACAGTTGCTGTTGCCTGTC-3′
TIMP1	TIMP1_490F	5′-AGGAATGCACAGTGTTTCCC-3′
	TIMP1_592R	5′-AAGCCCTTTTCAGAGCCTTG-3′
TIMP2	TIMP2_2430F	5-ACACGCAATGAAACCGAAGC-3′
	TIMP2_2503R	5′-AACAGGCTAAGGTGGCTTTG-3′
TIMP3	TIMP3_1700F	5′-TGCCTGCCTTGTACAAAAGC-3′
	TIMP3_1805R	5′-TGGCCAAATCTACCAAAGCG-3′
TIMP4	TIMP4_611F	5′-ACTGGCTGTTGGAACGAAAG-3′
	TIMP4_681R	5′-GCCGTCAACATGCTTCATACAG-3′
MMP2-AS1	MMP2-AS1_541F	5′-ATGTTGTGAGCAGCCCAATG-3′
	MMP2-AS1_682R	5′-TTGCCACTCAGCATCATCAC-3′
MMP23A	MMP23A_682F	5′-CCTCCACAAGAAAGGGAAAGTG-3′
	MMP23A_812R	5′-TGTAGGTGCCCTCATTGACG-3′
MMP24OS	MMP24OS_1029F	5′-ATCCCAGGGAAATGACACACTC-3′
	MMP24OS_1144R	5′-GGGACATCACAGCATTTCAGTG-3′
MMP25-AS1	MMP25-AS1_2017F	5′-AGTTCCGGAATGCAAAACCC-3′
	MMP25-AS1_2086R	5′-AGGACCTTGAAAGCATGTGG-3′
HAND2	hHAND2-Forward	5′-AGAGGAAGAAGGAGCTGAACGA-3′
	hHAND2-Reverse	5′-CGTCCGGCCTTTGGTTTT-3′
SNAI1	SNAI1_1405F	5′-TTTCAGCCTCCTGTTTGGTG-3′
	SNAI1_1489R	5′-TGACAGCCATTACTCACAGTCC-3′
EF1A	hEF1A_F	5′-TCTGGTTGGAATGGTGACAACATGC-3′
	hEF1A_R	5′-AGAGCTTCACTCAAAGCTTCATGG-3′

## Data Availability

The data that support the findings of this study are available from the corresponding author, (S.T.), upon reasonable request.
